# Thermo Compression of Thermoplastic Agar-Xanthan Gum-Carboxymethyl Cellulose Blend

**DOI:** 10.3390/polym13203472

**Published:** 2021-10-10

**Authors:** Smarak Bandyopadhyay, Tomáš Sáha, Daniel Sanétrník, Nabanita Saha, Petr Sáha

**Affiliations:** 1 Centre of Polymer Systems, University Institute, Tomas Bata University in Zlin, Tr. T. Bati 5678, 76001 Zlin, Czech Republic; dsanetrnik@utb.cz (D.S.); nabanita@utb.cz (N.S.); saha@utb.cz (P.S.); 2 Footwear Research Centre, University Institute, Tomas Bata University in Zlin, Nad Ovcirnou IV, 3685 Zlin, Czech Republic; tsaha@utb.cz; 3 Faculty of Technology, Tomas Bata University in Zlin, Vavrečkova 275, 76001 Zlin, Czech Republic

**Keywords:** thermo compression, blend, films, agar, xanthan gum, carboxymethyl cellulose, plastograph, food packaging, rheology, XRD, DTG

## Abstract

There is a gap in the literature for the preparation of agar-xanthan gum-carboxymethyl cellulose-based films by thermo compression methods. The present work aims to fill this gap by blending the polysaccharides in a plastograph and preparation of films under high pressure and temperature for a short duration of time. The pivotal aim of this work is also to know the effect of different mixing conditions on the physical, chemical, mechanical and thermal properties of the films. The films are assessed based on results from microscopic, infrared spectroscopic, permeability (WVTR), transmittance, mechanical, rheological and thermogravimetric analysis. The results revealed that the mixing volume and mixing duration had negative effects on the films’ transparency. WVTR was independent of the mixing conditions and ranged between 1078 and 1082 g/m^2^·d. The mixing RPM and mixing duration had a positive effect on the film tensile strength. The films from the blends mixed at higher RPM for a longer time gave elongation percentage up to 78%. Blending also altered the crystallinity and thermal behavior of the polysaccharides. The blend prepared at 80 RPM for 7 min and pressed at 140 °C showed better percent elongation and light barrier properties.

## 1. Introduction

Environmental pollution concerns have raised awareness and research regarding biodegradable polysaccharide-based food packaging material in the last decade [1,2]. Polysaccharide molecules are not only easily available and cheap, but they also form a continuous network by hydrogen bonding [3]. Among all the polysaccharides, agar [3], xanthan gum (XG) and carboxymethyl cellulose (CMC) [1] are reported widely as edible food packaging materials. Agar is a phycocolloid extracted from the cell wall of *Gelidium* sp. and *Gracilaria* sp. of Rhodophyceae red algae [4]. The molecular arrangement of agar is a combination of agarose and agaropectic. Agarose is formed by α-(1, 3) and β-(1, 4) glycosidic bonds between D-galactose and 3–6, anhydro-L-galactose; contrarily, agaropectin is slightly branched and sulfated [3]. Agarose is the gelling agent while agaropectin is the non-gelling agent removed during the industrial production of commercial agar. XG is a hetero polysaccharide produced by gram (-ve) *Xanthomonas campestris* during fermentation [5]. The chemical structure of XG consists of a d-glucopyranose glucan backbone linked with a β-(1, 4) glycosidic bond and α-(1, 3) trisaccharide side chain of mannose, glucuronic acid, and terminal mannose [6]. CMC is obtained by substitution (carboxymethylation) of –OH groups by sodium monochloroacetate in an alkaline medium [1]. The advantage of using CMC over native cellulose is the solubility in cold water due to its degree of substitution. The US food and drug administration (FDA) has also approved all the above-mentioned hydrocolloids as generally recognized as safe (GRAS).

A review of the literature showed no reports utilizing all three components (agar, XG and CMC) in a single blend for food packaging. When searching in Web of Science (WoS) with the keywords “agar” + “xanthan” + “CMC” + “blends”, there were only two articles found. One used them in gluten-free breads [7] and the other reported them as an interaction study with other food hydrocolloids [8]. Starch-based films are reported to have increased mechanical strength when blended with agar and XG by the solvent casting method [9]. The mixing of CMC with agar and gelatin exhibited improvements in barrier and mechanical properties [10]. Additionally, the addition of natural colorants to CMC-Agar blends gave a better water vapour barrier and strength to the films [11]. The addition of XG to the gelatin-CMC blend gave films with lower tensile strength but higher puncture resistivity [12]. Moreover, all the films made from the above composites use solvent casting methods. The work of Sousa et al. [13] has reported preparation of thermo compressed agar films with choline chloride and urea, but the films at higher temperature showed opacity. To the best of our knowledge, no work has been reported yet related to agar, XG and CMC films by thermal pressing methods.

The preparation of films by solution or solvent casting methods are well suited for laboratory scale production, but for industrial scaling-up utilization of instrumentation, it is a must. Moreover, methods like blown extrusion, compression or injection moulding of the agar, XG and CMC blends are also not reported, as far as we can tell. The casting methods require high energy and large amounts of time, thus creating space for tools designed for synthetic polymers. The extrusion, blowing, injection or thermo compression of the agar-based blends will not only make the throughput high but also energy efficient. In particular, thermo compression is advantageous for processing films because of its simplicity [14]. To mention in brief, when making films with thermo compression, the process starts and ends with simple mixing and pressing machine. In comparison to casting processes, these machines have a high throughput, reproducibility and save time.

Thus the aim of this study is to develop biodegradable transparent agar-based films by thermo compression. Furthermore, the effect of the blending conditions on the physical, chemical, mechanical and thermal properties of the films are also investigated.

## 2. Materials and Methods

### 2.1. Materials

The Agar used in this study was bought from HiMedia Laboratories Pvt. Ltd., Mumbai, India. The Xanthan gum from *Xanthomonas campestris* was procured from Sigma Life Science, Missouri, MI, USA, while the Sodium (6.5–8.5%) Carboxymethyl Cellulose was obtained from Sinopharm Chemical Reagent Co., Ltd., Ningbo, China. The Polyethylene Glycol 3000 (referred later as PEG) and Glycerol anhydrous GR were supplied by Fluka Chemie GmbH, Buchs, Switzerland and Lach-Ner s.r.o, Neratovice, Czechia respectively.

### 2.2. Preparation of Agar Based Thermoplastic Mixture

The components for mixing were divided into two parts: the solid (referred later as MS) and the liquid. The MS comprised of Agar (57.71%), XG (28.84%), CMC (7.69%), PEG (5.76%) and the liquid part were made of Glycerol and water mixed in a 3:1 ratio. The MS had a volume of approximately 1.44 cm^3^ per gram, while the liquid portion had a volume of approximately 0.92 cm^3^ per gram. This composition is chosen after much trial and error to fit the best mixing volume in the plastograph (data not shown in this article). The MS was premixed by shaking in a glass jar 30% filled and later mixed with the liquid portion in a Brabender R2400 Plastograph with W50 mixer (Brabender GmbH & Co KG, Duisburg, Germany). The volume of the mixer bowl was 55 cm^3^ and the mixing conditions are mention in Table 1.

The feel of the final blend by touch and its colour is mentioned in Table 2. Later, they were stored in plastic zip lock bags until further used for pressing.

### 2.3. Film Production by Thermo-Compression of the Mixture

**Figure 1:**
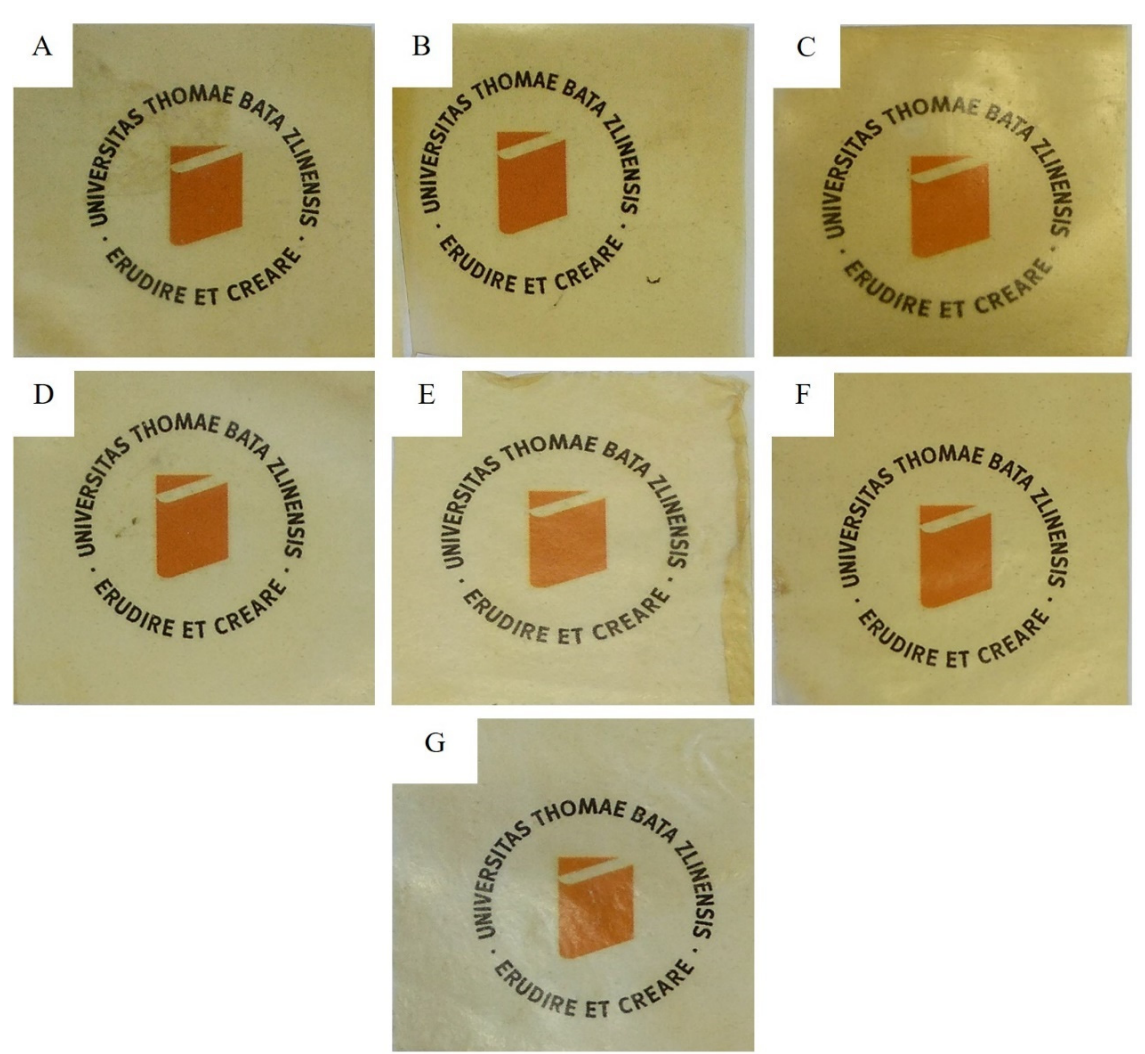
The films (**A**–**G**) after heat pressing placed over Univerzita Tomáše Bati ve Zlíně logo to show real transparency.

Thermal compression of 3.5 g from each blend was done between polytetrafluoroethylene (PTFE) coated fiberglass of 0.15 mm thickness. The steel frame used as a mold had dimensions of 130 mm × 130 mm × 4.1266 ± 0.007 mm, while the finally produced films had a length and breadth of 125 mm × 125 mm, with varying thicknesses as reported in Table 1. The films were prepared by pressing the blend for 3 min without pressure at 140 °C, then finally pressing for another 6 min at 140 °C with a constant pressure of 300 kN. The pressing temperature is kept way below the maximum degradation temperature (T_m_) of the individual components. The mixing time and pressure are selected from trial and error experiments (data not shown), with the best conditions selected for this study. The films were cooled under the same pressure in a cooling hydraulic press and then peeled off of the PTFE foils. The appearance of the films is shown in Figure 1.

### 2.4. Characterization of the Films

Scanning electron microscopy analysis was done using FEI™ (Nova Nano SEM, Oregon, Hillsboro, OR, USA). The surface of the films was gold-sputtered for 60 s at 30 mA. The images were captured at 10,000 magnification and 5 kV.

The Fourier transfer infrared (FTIR) spectroscopy and X-ray diffraction (XRD) was done following the exact method in our previous study [15]. The only change in XRD is the inclusion of the formula to convert Cobalt K alpha to Copper K alpha in OriginPro 8.5 (OriginLab Corporation, Northampton, MA, USA):

Cu K α = 114.59156*asin(L2/L1*sin(0.00872664*col(X)))

where, col(X) is the column having Co K α - 2θ value; L1 = 1.7891 (Co K alpha wavelength), L2 = 1.5418 (Cu K alpha wavelength).

The water vapour transmission rate (WVTR) of the materials was evaluated by the desiccant method following ASTM E96. Pre-dried silica beads at 150 °C [16] of 10 g weight were filled up to 1/3rd in a 50 mL glass beaker. The orifice of the beaker was covered with films and sealed with paraffin wax tape. The exposed area of the beaker and weight of the complete experiment setup (beaker with silica and film, referred further as cell) was noted. The cell was then placed inside a humidity chamber at 90% RH and 30 °C. A beaker with 100 mL water was also placed inside the chamber to note down the decrease in the water volume, absorbed by the cells each day. Three control cells were also placed without film cover, only having the 10 g silica beads. All the setups were triplicated for each film and the experiment was conducted for 6 days. Change in weight was recorded at 24 h intervals. The WVTR is calculated on the data for 24 h, using the formula [17]:

WVTR = (W_24_ − W_0_)/T × A

where W_24_ is the weight of the bottle after 24 h, W_0_ is the weight of the bottle at the start of the experiment, T is the time in days and A is the area in m^2^.

The water vapour absorption rate (WVAR) of the films is the representation of the data from the WVTR setup after 24 h. WVAR is the slope plot between the w% (change in weight of the cell from the initial weight, expressed in %) and time.

The light transmittance and film transparency was measured following the method of Alias et al. [18]. The films were cut into small pieces (4 cm × 1 cm) to fit inside the cuvette for reading with Cary 300 UV-visible spectrophotometer (Agilent, Santa Clara, CA, USA). All the films were first scanned from 200 to 800 nm wavelengths to select the peak for maximum absorption (A_max_). Later, the film transparency was calculated at 294 nm using formula [19]:

Transparency = A_294_/t

where A_294_ is the A_max_ or absorbance at 294 nm and t is the thickness of the films in mm.

The data of UV-Vis and measurement of thickness is an average of 5 replications. Additionally, the real degree of transparency is inversely proportional to the value of transparency obtained from the above equation.

The mechanical analysis was done as per the methods reported earlier in our study [20]. The cross-head speed was 10 mm/min and 10 kgf static load was used. The only change is the dimension of the samples to 15 mm × 2 mm.

The rheological analysis was done with modifications from our earlier report [15]. To mention in brief, the amplitude sweep was done with a strain range of 0.01–100% and angular frequency of 10 rad/s. The frequency sweeps were performed at 0.05% strain and angular frequency range from 0.01 to 100 rad/s. The slope of the curve is reported with 25 points per decade for amplitude sweep and 5 points per decade for frequency sweep. The experiment was conducted with a rough surface of 20 mm diameter parallel plate geometry at 25 °C.

Thermogravimetric analysis was performed as per our previous work [15]. Under a nitrogen environment (flow rate: 100 mL/min), the samples were heated at a rate of 10 °C/min between 25 and 600 °C.

### 2.5. Statistical Analysis

OriginPro 8.5 (OriginLab Corporation, Northampton, MA, USA) was used for statistical analysis, represent the result as Mean ± Standard Deviation with 5% error margin. The winTest Analysis 4.7.0 (Testometric Co. Ltd., Rochdale, UK) was also used for calculating the coefficient of variation (C. of V.). Design Expert ver 11 (Star-Ease Inc. Minneapolis, MN, USA) was used for two level factorial ANOVA analysis and optimization of WVTR data.

## 3. Results and Discussions

### 3.1. Morphology and Structure of Films

**Figure 2:**
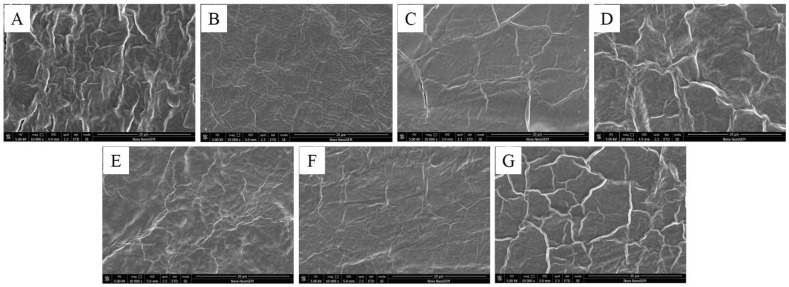
Surface SEM images of the heat pressed films (**A**–**G**).

The SEM images of the film’s surface are shown in Figure 2. All the films have only suggestive evidence of the components mixed well without showing any phase separation but the reticulated structure may be attributed to the presence of Agar in the films [21]. The structure is devoid of any pores on the surface and shows a homogeneous compact structure, formed may be due to the strong interaction among the newly formed bonds in all the films as evident from Figure 2. Glycerol has probably contributed to developing new intra and inter hydrogen bonds among the hydroxyl groups of the polysaccharide components by entering the interior of the polysaccharide components’ chains, breaking them and creating new ones [22].

**Figure 3:**
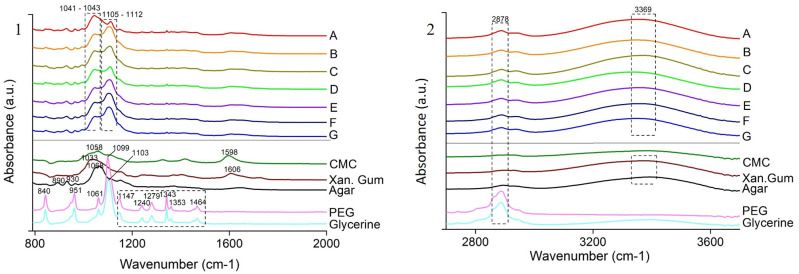
FTIR spectra of heat pressed films (A–G) and the individual components (CMC, Xanthan Gum, Agar, PEG and Glycerine) divided into two segments: (**1**) wavelength 800 to 2000 cm^−1^ (**2**) wavelength 2000 to 4000 cm^−1^.

The FTIR spectra in Figure 3 shows the characteristic peaks for all the individual components of the films, namely PEG and Glycerine [23,24,25] at 1099 cm^−1^ (C-H + O-H stretching), 1103 cm^−1^ (C-C + C-O-C stretching), 1279 cm^−1^ (O-H + C-O-H stretching), 1343 cm^−1^ (C-H bending), 1464 cm^−1^ (C-H bending) and 2878 cm^−1^ (C-H stretching); Agar [26] at 1068 cm^−1^ (C-O-C stretching); XG [27] at 1033 cm^−1^ (C-O stretching); CMC [28] at 1058 cm^−1^ (C-O stretching). The peak at 3369 cm^−1^ is due to the O-H stretching [29]. The shift in the peak of the individual polysaccharides from 1033–1068 cm^−1^ to 1041–1043 cm^−1^ and the addition of new peaks at 1105–1112 cm^−1^ in the blended films may suggest the formation of new interactions due to the addition of glycerine. The blend is a perfect amalgamation of all the individual components as it does not replicate the exact peaks as in the components, but shows different peaks with changed intensity due to the formation of new physical and chemical interactions [30].

**Figure 4:**
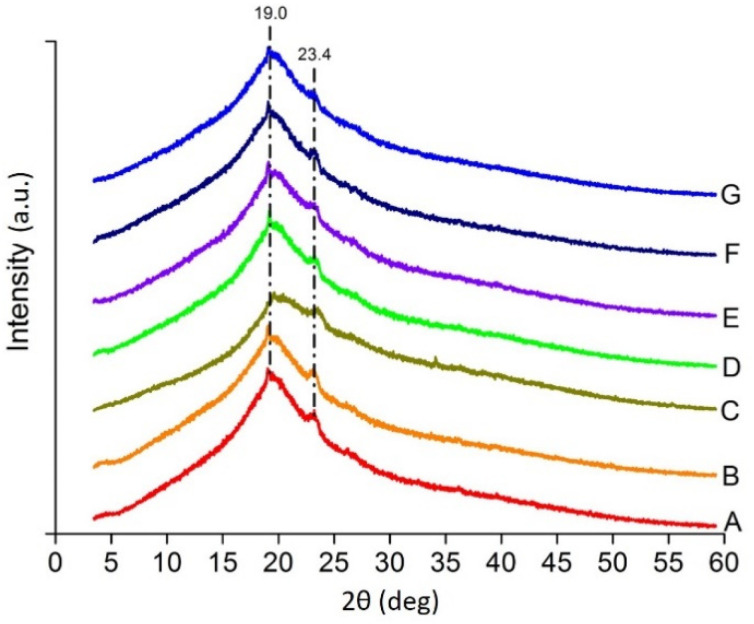
X-ray diffraction patterns of heat pressed films (A–G).

The XRD peaks of the blends are shown in Figure 4, which is similar to the peaks of the constituents, but not the same. The peak at 2θ = 19 is a near average from the constituents Agar at 19 [31], XG at 19.64 [32], CMC at 19.7 [33] and PEG at 19.23 [34] whereas 2θ = 23.4 may be because of PEG at 23.3 [34] or 23.4 [35] but with less intensity due to the mixing with polysaccharides. The other characteristic peaks of Agar at 14 and 27.2 [31]; XG at 16.42 and 20.10 [32]; CMC at 18.4, 22.7, 25.3 and 28.4 [33]; PEG at 13.6 and 27.3 [34] are missing due to decrease in the crystallinity of the final blend. The reduction in the crystalline property of the biopolymer blends which has occurred may be due to the mixing of the components [31].

### 3.2. Mechanical and Rheological Properties of the Films

The mechanical properties of the films were assayed by tensile testing at room temperature, reported in Table 3. The ε of the films A, B, D and F are better than the ε reported by different authors for pure casted films of Agar at 10% [36], 31% [37] and 45.2% [38]; XG at 56% [39]; CMC films at 50% [1] with glycerine as a plasticizer. The tensile strength of films A, B and F are nearly 40% of the strength of commercial cellulose acetate films [40].

The effect of mixing RPM and time on the mechanical properties is depicted by the variation in strength and elongation percentage of the films. The blends with a 4:3 ratio between MS and liquid, mixed at a higher RPM for a longer period of time, had improved tensile characteristics, perhaps due to good adhesion among the polysaccharides in the polymer matrix at the interface thus resulting in reinforcements [2].

**Figure 5:**
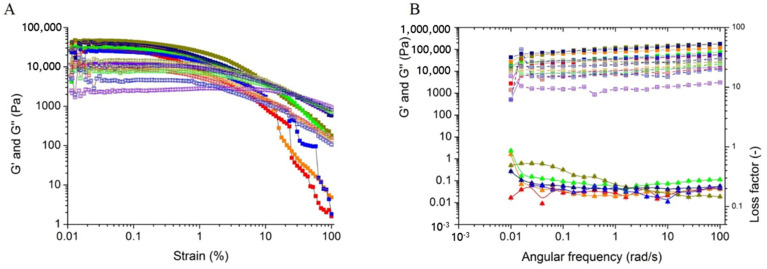
Rheological analysis of the heat pressed films, marked with distinct colors: A (Red), B (Orange), C (Dark Yellow), D (Green), E (Violet), F (Navy), and G (Blue). (**A**) Amplitude sweep with Storage modulus G’ (■) and Loss modulus (□); (**B**) Frequency sweep with Storage modulus G’ (■), Loss modulus (□) and Loss factor (▲) (The reader is requested to refer to the web version for better interpretation).

The amplitude sweep was done to determine the linear viscoelastic region (LVE region), reported in Figure 5A. Consequently, the amplitude sweep (Figure 5B) was done within the LVE region to avoid any sample deformation during measurements. The storage modulus of all the films is above the loss modulus for the entire frequency zone, thus suggesting the elastic behaviour of the material over viscosity. The MS content and mixing time had an effect on the viscoelastic properties of the films. As with a decrease in the solid portion and increase in mixing time, the viscoelasticity of the films changes from higher to lower. The reason behind the change may be due to the formation of better inter and intra hydrogen bonding among the polysaccharides [15] when mixed at high quantity for a lower time. As the elasticity and viscosity of the film C are greater than other films, so the same is reflected in the loss factor.

### 3.3. Physical Properties of the Films

Water vapour permeability is an important parameter for assessing the quality of food packaging material. WVTR decides the fate of the packaging material for packing suitable food products. In reports by other authors, WVTR of pure Agar and CMC cast films are reported to be 1130 g/m^2^.d [41] and 1098 g/m^2^.d [42], respectively.

Additionally, the addition of XG in higher amounts to hydrocolloids has been reported to reduce the water permeability of the films due to blocking of the free –OH groups (which binds with water vapour molecules) with new hydrogen bonds [9]. Our results show similar trends when the XG is mixed with Agar and CMC (Table 4).

**Figure 6:**
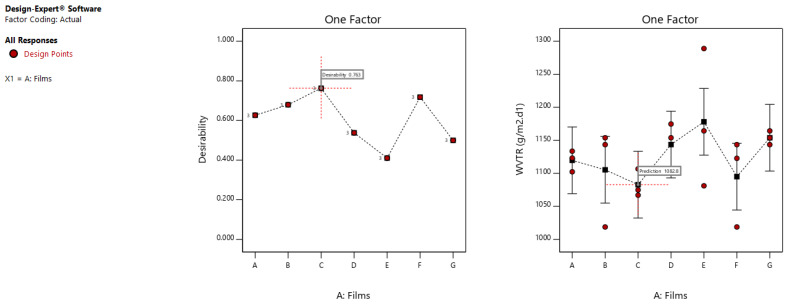
Optimization graph from two level factorial analysis for the WVTR of the films, showing the desirability and LSD bars interactions.

Statistically, the variability in mixing conditions had no effect on the vapour permeability. As shown in Figure 6, since the LSD bars overlaps with each other, the WVTR values do not have any statistically significant difference. The permeability obtained has a range from 1078 to 1082 g/m^2^.d^1^ (Table 4).

Thus, all the films show better WVTR than individual Agar and CMC films. The improvement in the barrier properties may be also due to the presence of polysaccharides at higher concentrations which results in thicker compacted matrix arrangement with high solid concentration/area [9].

PEG and Glycerol may not have affected the WVTR when the polysaccharides are present in higher concentrations (≈75%) in the films, as not being able to open the strong interactions in the network structure of the blend in a continuous phase [41].

**Figure 7:**
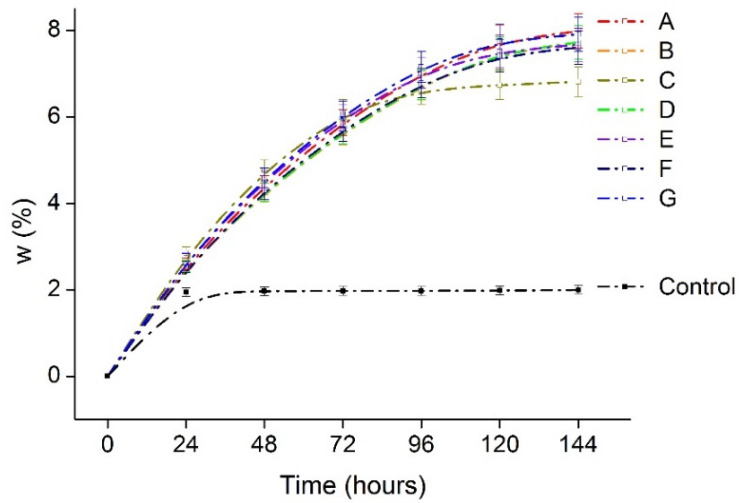
Water vapour absorption rate of the heat pressed films A–G in 90% RH humidity chamber.

Figure 7, shows the WVAR or moisture absorption capacity of the films in high humid conditions. It is evident from the graph that the films keep on absorbing moisture from the environment until 120 h, in contrast to the control with only silica beads reached saturation after 24 h. In real applications, Agar-XG-CMC films can also lower the water activity (A_W_) in the package and prevent microbial spoilage. As suggested by Nur Hazirah et al. [12] from their study, films with higher barrier properties than conventional packaging materials may be used where WVTR is not essential for the foods packed in it or a secondary packaging can be given with desirable barrier properties. Additionally, moisture absorbing films can also be used as a moisture triggered active packaging material [43].

**Figure 8:**
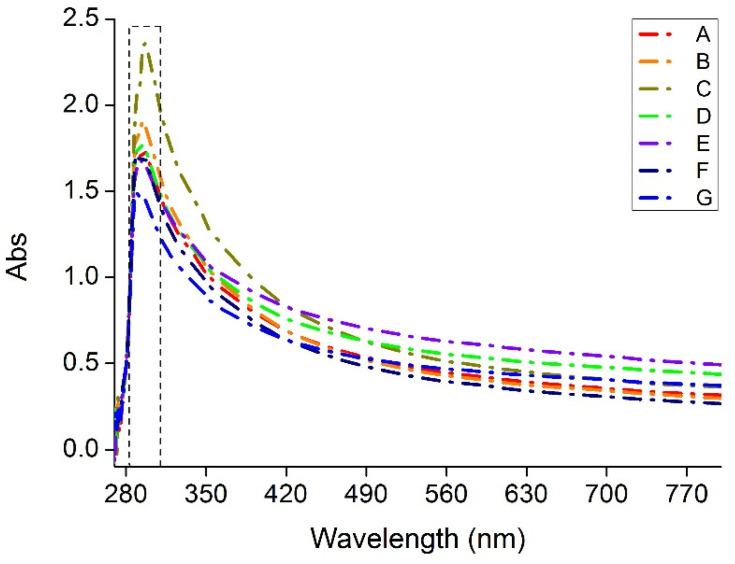
Absorbance of the heat pressed films (A–G) from wavelength 200 nm to 800 nm.

Ultraviolet-C (UV-C: 100 to 280 nm) is recommended and used for the sterilization of food against bacteria and viruses [44,45]. As shown in Figure 8, all the films show maximum absorption with wavelengths between 280 and 300 nm, so as suggested by Calle et al. [44], wavelengths in the range 250 to 280 nm can be used to sterilize food contents when packed with Agar-XG-CMC films.

Since the transparency of the films was calculated on absorbance, the value of transparency mentioned in Table 3 is inversely proportional to the actual degree of UV transparency. The mixing time has an effect on the transparency of the films; lowering mixing time irrespective of RPM gives films with better transparency and lower transparency with increasing time. Mixing the blend for a longer time may result in oxidative degradation of the polymeric chains which in turn affects the colour of the blend and overall transparency. Film C has the best barrier against UV and Film E for visible light.

### 3.4. Thermal Properties of the Films

**Figure 9:**
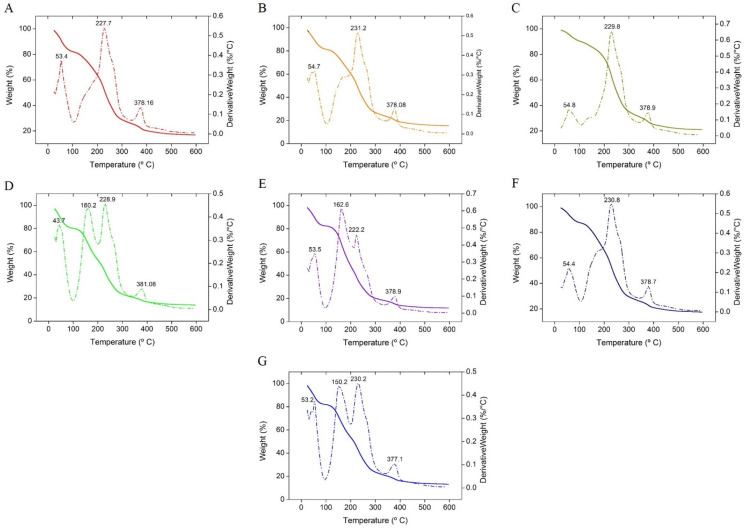
TGA and DTG signals of heat pressed films (**A**–**G**).

Figure 9A–G shows the TG and DTG curves of the Agar-XG-CMC-based films from room temperature to 600 °C. The degradation occurs in three to four steps. In the first phase below 100 °C, the evaporation of water vapour takes place, resulting in 20% mass loss. The second phase marked by T_m_ in DTG from 150 °C to 160 °C in films D (Figure 9D), E (Figure 9E) and G (Figure 9G) are due to reminiscent from the excess plasticizers PEG + glycerol [46,47] which may not have bonded with the hydrocolloids. The films with ≥50% liquid share during mixing show the second degradation peak between 150 and 160 °C. The change in mass of all the films is negligible from the start of the second phase until the start of the third phase but the third phase with T_m_ from 227.7 °C to 230.2 °C ends with degradation of 80% mass. The fourth phase is marked with T_m_ from 377.1 °C to 381.08 °C. The T_m_ in the third and the fourth phase may be due to the amalgamation of the pure polysaccharides with T_m_ reported for Agar at 300 °C [48], XG at 290 °C [49] and Na-CMC at 280.89 °C [50]. Thus, it is also evident that the mixing and compression temperatures are kept far below the degradation temperatures.

## 4. Conclusions

The transparency of the films was negatively dependent on the mixing volume and mixing time respectively. The mixing conditions had no effect on the WVTR. The tensile strength of the films was positively dependent on the mixing RPM and mixing time. The crystallinity and thermal behaviour of the individual polysaccharides changed due to blending. The films in our study showed more elongation when prepared by blending followed with thermal pressing than casting of the individual components as reported by previous works. A direct comparison between blending and casting cannot be drawn unless they are done with the same composition; thus, this is a scope for future research. Moreover, it can be concluded that since different blending conditions have an effect on the properties of the films, industry prototypes of agar blends can be developed with blending techniques. The improvement in the elongation at break suggests the use of these materials, especially film F, as a primary wrapping material for less moisture sensitive foods. The suggested blending conditions of Agar blends for future use may be mixing at 80 RPM for 7 min with 4:3 ratio between solid to liquid and 84% working volume, 16% free volume.

## Figures and Tables

**Table 1:** The mixing conditions of the heat pressed films.

Components	Film A	Film B	Film C	Film D	Film E	Film F	Film G
RPM	100	100	100	100	80	80	80
MS, g	26	20	20	10	20	20	10
Liquid, mL	20	15	10	10	30	15	10
Time, min	10	7	5	5	7	7	5
Total mixing volume, cm^3^	50.02	42.74	38.13	23.67	56.54	42.74	23.67

**Table 2:** The feel, visual appearance (colour) of the blends after mixing in a plastograph.

Blends for Films:	A	B	C	D	E	F	G
Softness/Hardness ^1^	4	5	6	2	0	3	1
Visual appearance/Colour ^2^	5	4	2	1	3	4	0
Remarks ^3^	-	-	-	-	Films formed from this blend were sticky	-	Films formed were less sticky than E

^1^ 0 = Maximum soft; 6 = Maximum hard; ^2^ 0 = Maximum white; 5 = Maximum brown; ^3^—Normal films without stickiness.

**Table 3:** Mechanical properties of the heat pressed films (E = Young’s Modulus, σ = Tensile strength and ε = Elongation at break).

Components	Film A	Film B	Film C	Film D	Film E	Film F	Film G
E, MPa ^1^	9.91 ± 3.50 _35.4_	7.50 ± 3.13 _41.81_	183.22 ± 64.5 _35.23_	8.62 ± 4.23 _49.13_	4.12 ± 0.88 _21.44_	8.76 ± 3.27 _37.36_	5.46 ± 0.99 _18.21_
							
σ, MPa ^1^	0.59 ± 0.21 _36.58_	0.59 ± 0.08 _14.43_	0.49 ± 0.03 _6.97_	0.33 ± 0.04 _12.95_	0.10 ± 0.03 _27.5_	0.61 ± 0.04 _8.01_	0.07 ± 0.02 _33.1_
							
ε, % ^1^	73.75 ± 19.05 _25.8_	78.40 ± 14.75 _18.8_	16.40 ± 9.36 _57.08_	62.85 ± 14.05 _22.3_	32.39 ± 13.14 _40.5_	73.40 ± 21.20 _28.8_	28.38 ± 2.31 _8.13_

^1^ Mean ± Std. Dev. _C. of V_.

**Table 4:** Water vapour transmission rate, transparency, water content and thickness of the heat pressed films.

	Film A	Film B	Film C	Film D	Film E	Film F	Film G
WVTR, g/m^2^.d ^1^	1119.6 ^a^ ± 15.8 _1.41_	1105.3 ^a^ ± 75.19 _6.80_	1082.8 ^a^ ± 21.06 _1.94_	1143.5 ^a^ ± 37.2 _3.25_	1178.1 ^a^ ± 104.6 _8.88_	1094.9 ^a^ ± 66.8 _6.10_	1153.8 ^a^ ± 10.3 _0.90_
							
Transparency ^1^	16.18 ± 1.33 _8.26_	16.14 ± 1.39 _8.62_	13.80 ± 0.43 _3.12_	12.01 ± 0.99 _8.24_	14.91 ± 0.98 _6.57_	16.98 ± 3.41 _20.08_	11.95 ± 0.42 _3.52_
							
Moisture % ^1^	6.34 ± 0.1 _1.65_	7.24 ±5.11 _70.65_	5.84 ±0.39 _6.73_	10.05 ± 1.8 _8.69_	10.83 ± 0.57 _5.29_	6.26 ± 0.29 _4.73_	7.69 ± 0.62 _8.06_
							
Thickness, mm ^1^	0.095 ± 0.008 _8.32_	0.099 ± 0.008 _8.37_	0.141 ± 0.005 _3.25_	0.125 ± 0.009 _7.56_	0.099 ± 0.007 _6.69_	0.085 ± 0.022 _26.3_	0.107 ± 0.004 _3.45_
							

^1^ Mean ± Std. Dev. _C. of V._; ^a^ Statistically insignificant at *p* = 0.05.
